# Flexible Electromagnetic Sensor with Inkjet-Printed Silver Nanoparticles on PET Substrate for Chemical and Biomedical Applications

**DOI:** 10.3390/s24206526

**Published:** 2024-10-10

**Authors:** Muhammad Usman Ejaz, Tayyaba Irum, Muhammad Qamar, Akram Alomainy

**Affiliations:** 1 Dyson Institute of Engineering and Technology, Malmesbury SN16 0RP, UK; 2 School of Electronic Engineering and Computer Science, Queen Mary University of London, London E1 4NS, UK; t.irum@qmul.ac.uk (T.I.); m.qamar@qmul.ac.uk (M.Q.)

**Keywords:** flexible, hydrocortisone, metamaterials, PET substrate

## Abstract

For this article, a low-cost, compact, and flexible inkjet-printed electromagnetic sensor was investigated for its chemical and biomedical applications. The investigated sensor design was used to estimate variations in the concentration of chemicals (ethanol and methanol) and biochemicals (hydrocortisone—a chemical derivative of cortisol, a biomarker of stress and cardiovascular effects). The proposed design’s sensitivity was further improved by carefully choosing the frequency range (0.5–4 GHz), so that the analyzed samples showed approximately linear variations in their dielectric properties. The dielectric properties were measured using a vector network analyzer (VNA) and an Agilent 85070E Dielectric Probe Kit. The sensor design had a resonant frequency at 2.2 GHz when investigated without samples, and a consistent shift in resonant frequency was observed, with variation in the concentrations of the investigated chemicals. The sensitivity of the designed sensor is decent and is comparable to its non-flexible counterparts. Furthermore, the simulation and measured results were in agreement and were comparable to similar investigated sensor prototypes based on non-flexible Rogers substrates (Rogers RO4003C) and Rogers Droid/RT 5880), demonstrating true potential for chemical, biomedical applications, and healthcare.

## 1. Introduction

**Figure 1:**
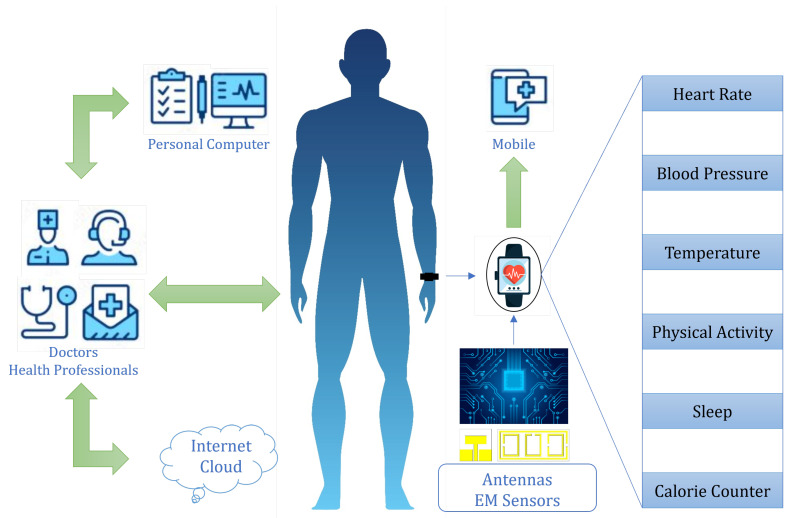
Role of antennas and EM sensors in biomedical applications and wireless health monitoring.

Electromagnetic devices, such as antennas, lenses, resonators, absorbers, and sensors, have been extensively investigated for their diverse applications in the communication, defense, security, chemical, and biomedical industries [1]. Antennas, resonators, and sensors are key components of sensing systems in the chemical and biomedical industries. Demand for these sensing systems has risen substantially, predominantly for concentration estimation and for monitoring of important industrial-use chemicals, early diagnostics of diseases, and monitoring of numerous physiological parameters [2]. EM-based sensing devices are promising for divergent applications, due to their high speed, cost-effectiveness, simplicity, high sensitivity and selectivity, and non-destructive nature [3]. The emerging interest in flexible materials has led to the development of soft, conformal, and biocompatible antenna systems, facilitating the impeccable integration of these systems in bioelectronics. Bioelectronics technology is one of the most influential and driving aspects of electronic devices that are designed to operate in the vicinity of the human body. Extensive research and development have been conducted, to make flexible, small, cost-effective, and biocompatible sensing devices for a variety of applications in healthcare and medical diagnostics. A wireless electronic sensing and monitoring device can provide the opportunity for real-time monitoring and processing of various bodily parameters and signals associated with different measurements, as illustrated in Figure 1. A specific frequency band (the Industrial, Scientific, and Medical (ISM) radio band) is dedicated and reserved internationally for the use of electromagnetic devices in various ISM applications [4].

Electromagnetic sensors are governed by the working principle of change in the resonant frequency and quality factor of the prospective design. The dimensions are determined by the desired operating frequency, electrical permittivity ($\epsilon_{r}$), and magnetic permeability ($\mu_{r}$). The sample under investigation is either introduced inside the sensor structure or in close proximity to the sensing device. The interaction of the sample with the electric field is maximum, and variation in electrical permittivity causes a change in the reflective index n, which is given as [5]

(1) $$n=\pm \sqrt{\epsilon_{r}\mu_{r}}$$

Here, $\epsilon_{r}$ and $\mu_{r}$ are the relative electrical permittivity and the magnetic permeability, respectively. When the sample under investigation is introduced into the sensing device, a change in electromagnetic boundary conditions occurs, resulting in resonant-frequency and quality-factor shifts. Most resonators or sensors are implemented with planar techniques in the literature. These sensors are based on SRR (split-ring resonator) topology, micro-strip-line resonators, and patch antennas. In general, these sensing devices have higher accuracy and sensitivity, as the measurement principle is based on a shift in resonant frequency or a variation in the quality factor of the given design [6,7]. A complete sensor structure with split-ring resonators can be regarded as a series of RLC resonance circuits, for which the resonance frequency can be formalized as [7]

(2) $$f=\frac{1}{2\pi \sqrt{LC}}$$

### 1.1. Chemical Applications

Innovative microfluidic chemical sensors are quite prominent among other electromagnetic sensors. Methodical selection and formulation of design parameters have led to the implementation of versatile electromagnetic sensors having comprehensive application in the microwave-frequency and millimeter-wave-frequency domains. The dielectric characteristics and frequency responses of such sensors are sensitive to temperature, density, humidity, strain, and pressure variations. These variations can be interpreted by analyzing and comparing the frequency responses at the initial and final states of measurement [8,9,10].

Rahani (2011) introduced the idea of utilizing EM sensors to detect heat-induced damage in different dielectric and composite materials. The damage was calculated by sensing the variation of dielectric properties upon exposure to heat. It was concluded that the dielectric properties of materials are highly influenced by exposure to heat or high temperatures. Dielectric properties are described by complex electrical permittivity (ϵ = $\epsilon^{\prime }$ – i$\epsilon^{\prime \prime }$) and complex magnetic permeability (μ = $\mu^{\prime }$ – i$\mu^{\prime \prime }$), where real parts participate in the shift in resonance frequency and imaginary parts participate in resistive losses [11]. Withayachumnankul et al. (2013) investigated an electromagnetic sensor utilized for microfluidic measurements. The resonance frequency and bandwidth were affected by the change in the complex electrical permittivity of various liquid samples [12]. Ebrahimi et al. (2015) designed an EM-based microfluidic sensor operating in the microwave frequency range to determine the concentration of glucose in water-based solutions [13]. Zarifi et al. (2015) investigated microwave sensors comprising microwave resonators coated with a layer of PDMS (polydimethylsiloxane) acting as a sorbent layer that distended with the absorption of acetone, consequently changing its electrical permittivity and magnetic permeability [14]. Altintas et al. (2017) investigated a metamaterial-based sensor for concentration estimation of ethanol, strain, and rotation-sensing measurements [15]. Mehmet Bakir (2017) investigated an EM sensor made up of a split-ring resonator (SRR) coupled with a microstrip line for microfluidic applications. The sensor was utilized for concentration estimation of ethanol, methanol, acetone, ammonia, and polyethylene glycol (PEG 300, PEG 400) in corresponding aqueous solutions [16]. Hayder et al. (2018) designed a split-ring resonator-based electromagnetic sensor operating at 2.5 GHz, to differentiate between different pH levels, where hydrochloric acid (HCl) and sodium hydroxide (NaOH) were used to vary the pH of the solution samples [17]. André Soffiatti (2018) investigated a couple of patch antennas to estimate the concentration of two chemicals (sodium chloride (NaCl) and ethanoic acid ($C_{2}H_{4}O_{2}$)in aqueous solutions. The transmission coefficient was measured between two antennas, and a significant increase in transmission (dB) was observed with an increase in the concentration of chemicals in corresponding solution samples at four different frequencies (1.4 GHz, 2.4 GHz, 3.4 GHz, and 4.1 GHz) [18]. Ejaz et al. (2020) designed and investigated a versatile multi-layered metamaterial-based sensor operating in an X-band frequency (8–12 GHz) for chemical, biomedical, and strain sensing. Methanol and edible-fat solution samples were investigated for concentration sensing [19]. Javed et al. (2020) proposed a low-cost, non-invasive, and convenient-to-fabricate split-ring resonator-based sensor design fabricated on an FR4 substrate for dielectric characterization of liquids including ethanol and methanol mixtures [20]. Dalgac et al. (2021) investigated a metamaterial-based transmission-line sensor structure, to determine methanol contamination in various local spirits [21]. Bagci et al. (2022) investigated a metamaterial-based flexible sensor design based on a polycarbonate substrate for microfluidic measurements of ethanol and methanol mixtures [22].

### 1.2. Biomedical Applications and Healthcare

Prodigious research interest lies in the modeling and realization of the dielectric property measurements at microwave and millimeter-wave frequencies for biological tissues and biomaterials, such as skin, blood, fat, cholesterol, cerebral tissue, and intestine tissue. Extensive effort is essential to developing dielectric models of various biomaterials and tissues, to progress in this domain. The sensing potential and capability of EM-based biosensing devices in the detection of biochemicals and biomaterials are crucially dependent on dielectric properties like electrical permittivity, which are manipulated by different constituents of biological samples like cells, proteins, and DNA (deoxyribonucleic acid). Spada et al. (2011) proposed a metamaterial-based biosensor to detect malignant cancer and tumors. The proposed metamaterial-based sensor could conveniently differentiate between healthy and malignant tissues [4]. The evolution of EM-based biomedical sensing devices ameliorates the track of accurate monitoring of various physiological parameters like heart rate, pulse, stress, sweat, and blood pressure for medical diagnostics and curative treatment. A remarkable benefit of using microwave sensors is that they are adequate for contactless and non-invasive measurements while minimizing the associated destruction by short-distance measurements using penetrating waves. The non-ionizing nature of EM waves eliminates the chance of severe health hazards associated with other counterparts (like X-rays), particularly in biomedical sensing and healthcare [23,24,25,26,27].

**Figure 2:**
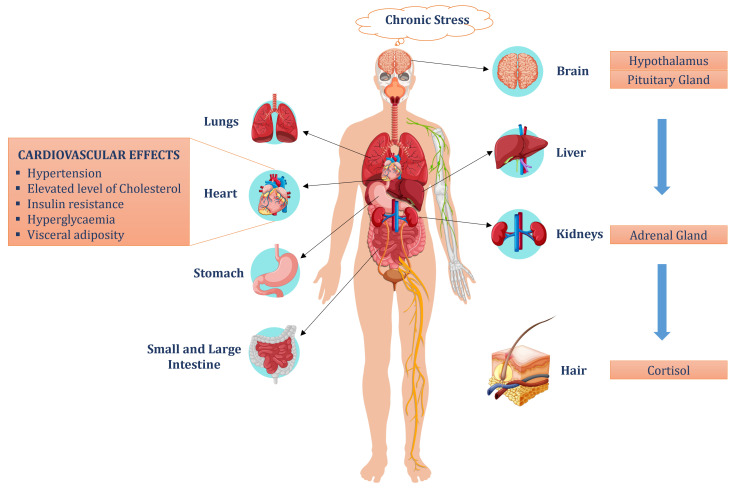
Mechanism of rise in hair cortisol with the increase in chronic stress and its cardiovascular effects.

Cardiovascular diseases (CVDs) are prime contributors to elevating mortality rates and augmenting health complications worldwide. There are numerous factors accountable for the consequential growth of cardiovascular diseases, like chronic stress, high blood pressure, high cholesterol, obesity, physical inactivity, and diabetes. Recently, the investigation of the role of chronic stress in cardiovascular diseases is attracting significant attention, due to its catastrophic effects on both mental and physical health. Negative emotional states like stress, anxiety, and depression can trigger cardiovascular effects independently of classical risk factors (hypertension, adiposity, diabetes, and physical inactivity), further escalating the risk of cardiovascular diseases [28,29,30]. The HPA axis (hypothalamic pituitary adrenal axis) is a fundamental stress-response system in humans, with the paramount function of facilitating successful adaptation and maintaining equilibrium with the external environment. The HPA axis activates in response to chronic stress, sending a message to the hypothalamus, further stimulating the production of adrenocorticotropic hormones (ACTH) from the pituitary gland. These hormones prompt the adrenal gland in the kidneys to release glucocorticoids including cortisol (the end product of this cycle). An elevated level of cortisol contributes to various health conditions, like hypertension, insulin resistance, hyperglycemia, and obesity. The cardiovascular effects of chronic cortisol exposure can be evaluated by cortisol measurements in body fluids (saliva, blood, sweat, and urine) and human hair [28,29,30,31]. The process of cortisol development and its effects are illustrated in Figure 2. Measurement of cortisol in hair offers various advantages over other specimens, being a non-invasive, low burden, and single-sample measurement. Hair samples are collected from the posterior side of the head, close to the scalp, and methanol is added, to extract cortisol during an overnight incubation (16 h) at 52 °C. The methanol is then transferred into a clean glass container and is evaporated until completely dry. The samples are then dissolved in PBS (phosphate buffer saline) with a pH value of 8.0 and mixed thoroughly. Cortisol levels in the hair extracts are then measured, using a commercial kit, also used for measurement of salivary cortisol in which a cortisol standard is available to generate a standard curve for the assay, and all samples are read off a user-generated standard curve [28,29,30,31].

**Figure 3:**
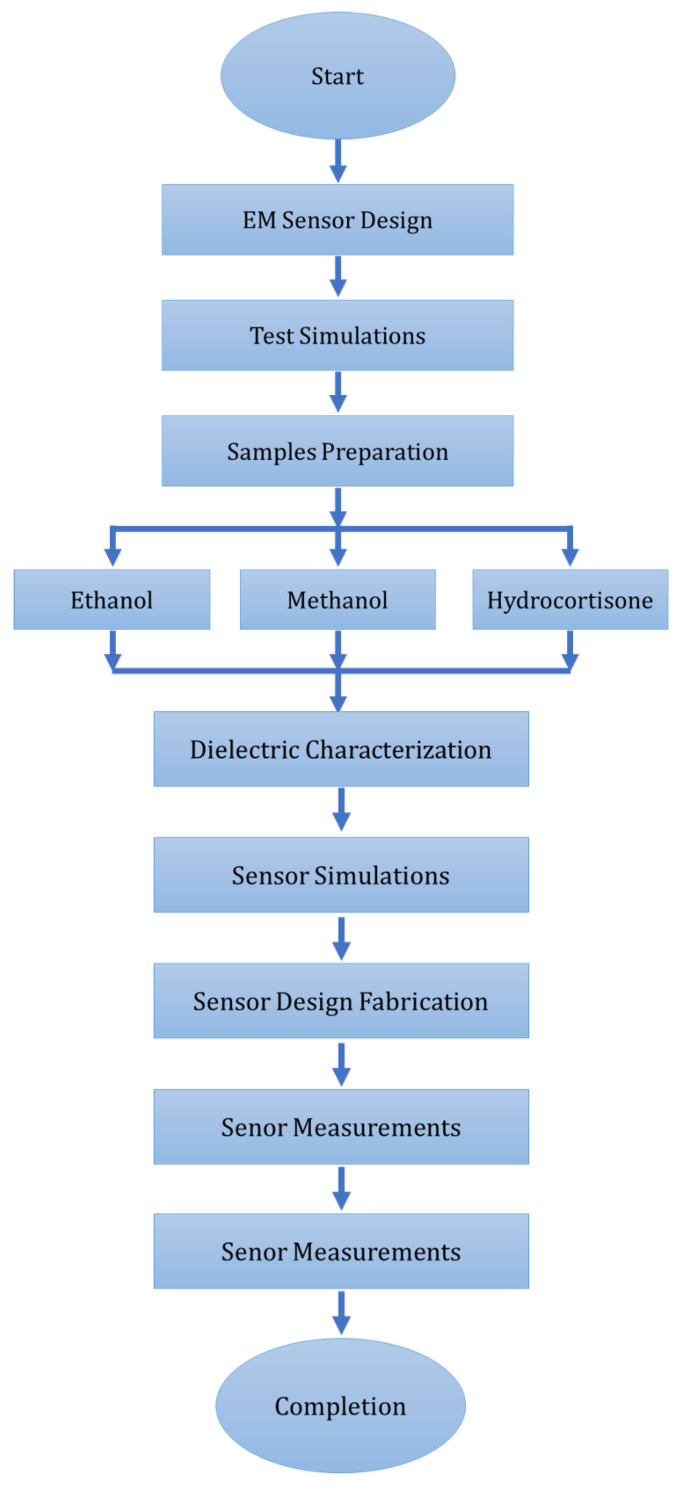
Design flow chart for sensor under investigation.

In short, numerous novel configurations of the microwave resonator and sensors have been investigated for microfluidic chemical and healthcare applications. Cavity resonator-based sensing devices have been investigated, to address the obstacle of lower accuracy, but these devices are expensive and require complicated experimental setups, making them incompatible with practical industry applications [32,33,34]. Dielectric characterization has been achieved, using complementary split-ring resonators (CSRR) coupled with a microstrip line, where liquid samples are contained in a polydimethylsiloxane (PDMS) channel in the sensitive region of a sensing device. Microfluidic channels built using polymers have been extensively investigated for dielectric characterization and liquid sensing, but fabrication and integration of these channels requires a complicated and delicate procedure for better results [35,36,37]. The submersible technique has been proposed in various investigations, in which submersible resonator structures are utilized for dielectric characterization and liquid sensing in the microwave frequency regime [38,39,40]. SRR and CSRR topologies in sensing devices are deterministic and practical for microfluidic sensing and for chemical and biomedical applications, due to their distinctive features and unique resonance characteristics [32,33,34,35,36,37,38,39,40]. D. Barmpakos et al. investigated graphene-based inks for flexible inkjet printed sensors, for thermal applications with high repeatability and endurance in heating cycles. The approach was promising for fabrication on non-planar substrates for selectively heated geometries [41]. J. George et al. proposed an inkjet-printed electromagnetic sensor with silver ink and carbon nanotubes as conducting material fabricated on a paper substrate. The design was validated for a differential gas sensor based on variations in dielectric properties of sensitive composite material in the presence of ethanol vapors [42]. H. Jeong et al. investigated a polarization-insensitive optically transparent metamaterial absorber fabricated on a PET substrate using inkjet printing with 90% absorption happening in the bandwidth of 26.8 to 28.2 GHz [43]. In contrast to various methods of cortisol measurement discussed in the literature, an economical, compact, and flexible electromagnetic sensor was proposed for the measurement of hydrocortisone (chemical replacement of cortisol hormone from Sigma-Aldrich, regarded as a biomarker of chronic stress in cardiovascular diseases) along with concentration measurements of two chemicals (ethanol and methanol) by examining the variation in dielectric properties and changes in resonant frequency in response to change in concentration. Dielectric characterization of various solution samples was conducted, using a network analyzer and a dielectric probe kit. These dielectric measurements were further utilized to investigate and validate the sensitivity of the proposed sensor design in simulation and experimental measurements. A shift in the resonant-frequency metamaterial-based sensing structure distinguishes significant variation in dielectric properties. Outlined in Figure 3 is the flowchart illustrating the design process followed for the proposed sensor.

## 2. Sensor Design and Fabrication

**Figure 4:**
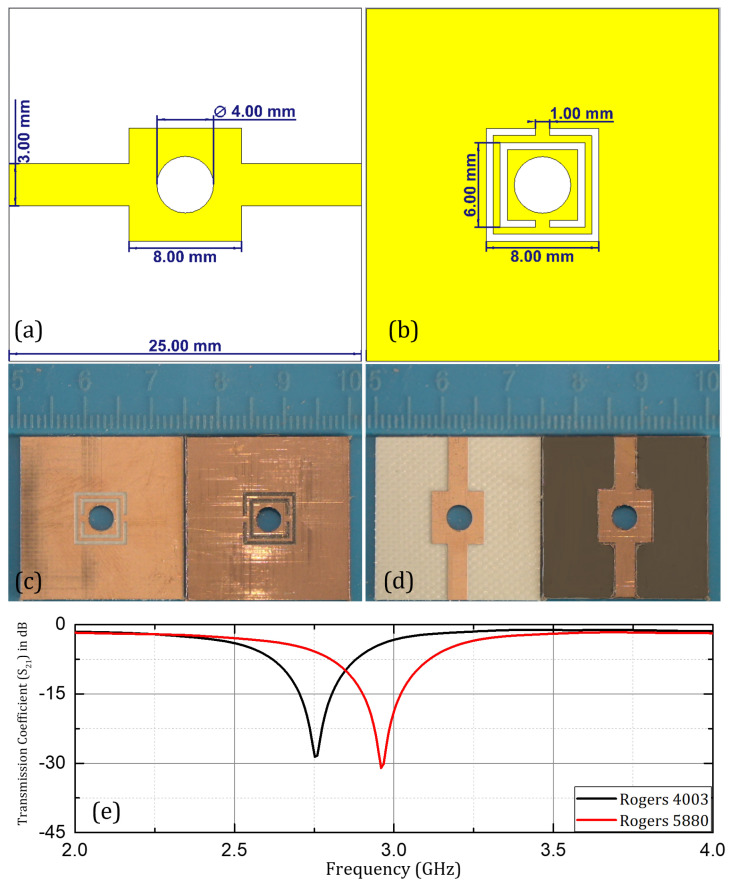
Proposed design: (**a**,**b**) front and back view with dimensions; (**c**,**d**) fabricated design; (**e**) measured transmission coefficient ($S_{21}$).

Microwave sensing has a diverse range of applications, due to its non-invasive and non-destructive nature, which include dielectric characterization, microfluidic sensing, health monitoring, biomedical sensing, and healthcare. An economical, compact, flexible, and contactless metamaterial-based microwave sensor for microfluidic applications is proposed here. The sensor structure is based on split-ring resonators made on three different substrates: Rogers RO4003C, Rogers RT/Duroid 5880, and flexible PET (polyethylene terephthalate). The sensor operates in the frequency range of 2.2–3 GHz without a sample, shifting to various resonant-frequency peaks corresponding to various samples introduced in the sample region with a high electric field. The purpose of investigating three different substrates is to compare the sensing performance of the non-flexible substrates (Rogers RO4003C and Rogers RT/Duroid 5880) with the flexible PET (polyethylene terephthalate) substrate. Roger RO4003C has a dielectric constant (ϵr) value of 3.38, a loss tangent value of 0.0027, and a thickness of 0.8255 mm. Roger RT5880 has a dielectric constant of 2.2, a loss tangent value of 0.0009, and a thickness of 0.787 mm. Both substrates have copper cladding on both sides with a thickness of 0.035 mm and conductivity of 5.8 × 10^6^ S/m. A microstrip line is carved on the front face of the design, while two square-shaped split-ring resonators (SSRRs) are etched into the ground plane on the backside. A profile view of the complete sensor design (with Rogers’ substrates) with dimensions has been shown in Figure 4. Sensor designs based on Rogers RO4003C and on Rogers RT/Duroid 5880 are fabricated using LPKF computer numerical control (CNC) prototyping machine.

**Figure 5:**
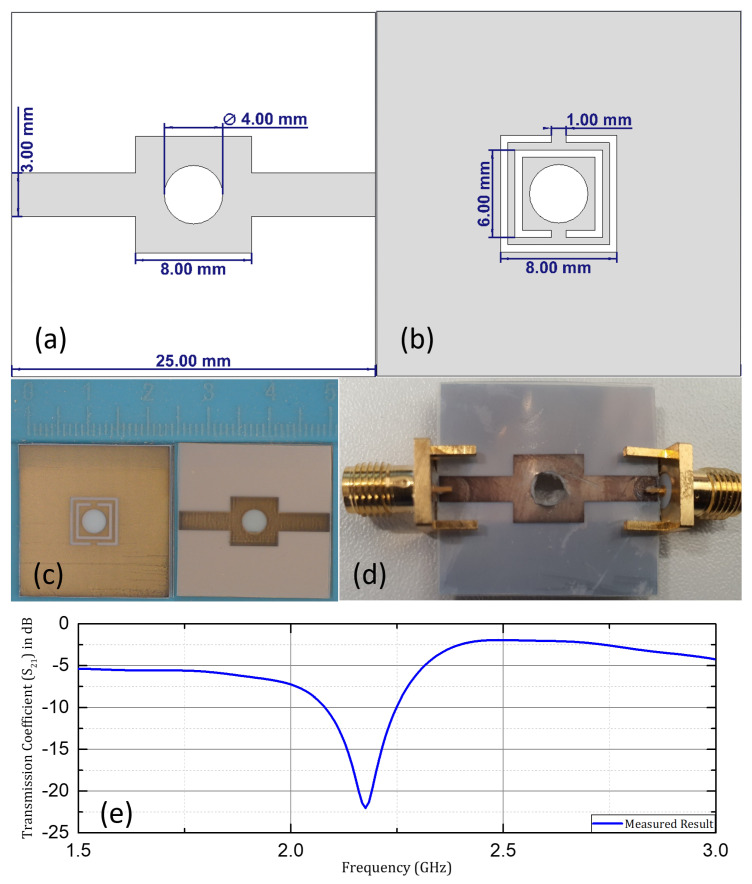
Proposed design fabricated on a PET substrate: (**a**,**b**) front and back view with dimensions; (**c**,**d**) fabricated design; (**e**) measured transmission coefficient ($S_{21}$).

The same sensor design is simulated and fabricated on PET film with a treated surface for better adherence to the conducting layer. The selected PET layer has a dielectric constant ($\epsilon_{r}$) value of 3.2, loss tangent (tanδ) value of 0.002, and the substrate height is kept at 0.3 mm. A Dimatix materials printer (DMP-2831) is employed for inkjet printing of a thin conductive layer of silver (silver nanoparticle ink with a conductivity of 0.4–2.5 × 10^7^ S/m), with an approximate thickness of 0.4–0.6 μm, and with the pattern resolution of around ±20 μm. Various parameters should be taken into consideration for primary setup, to achieve accurate calibration by the inkjet printer, including the jet frequency, drop spacing, head temperature, firing voltage of ejecting nozzles, and waveform characteristics. These parameters should be calibrated accurately, to achieve the best results in fabrication [44]. Silver nanoparticle ink used for fabrication consists of silver particles dispersed in an inert solvent and encapsulated in polymers to prevent oxidation. Curing, sintering, and drying processes are inevitable, to supplement the conductivity of the fabricated design. A drop spacing of 15 μm (1693.33 dpi) is selected for high-quality printing and optimum results. Conductivity can be manipulated by the number of printed layers, the temperature, and the sintering process. Conductivity in the range of 0.3–0.7 × 10^7^ S/m can be accomplished with a single printed layer if all the primary parameters are calibrated precisely, with proper curing and sintering of the design after fabrication [45]. SMA (subminiature version A) connectors are connected to the microstrip and ground conductor, using conductive epoxy (CircuitWorks^®^). It is based on silver, with two components mixed in the same ratio, providing a strong bond and excellent conductivity. The dimensions of the sensor structure are optimized in the CST microwave studio program, used for simulations. The simulated and fabricated design with the dimensions is shown in Figure 4 and Figure 5.

**Figure 6:**
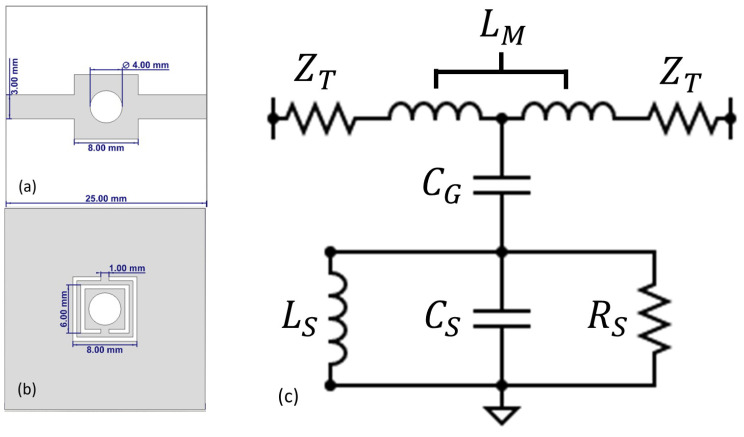
PET based sensor design with equivalent circuit model.(**a**,**b**) front and back view with dimensions; (**c**) equivalent circuit model ($L_{S}$ = inductance of SRR, $C_{S}$ = capacitance of SRR, $R_{S}$ = resistance of SRR, $C_{G}$ = capacitance between ground plane and microstrip line, $Z_{T}$ = Impedance of microstrip line, $L_{M}$ = inductance of microstrip line).

The proposed sensor structure comprises two square-shaped resonator rings with a defined gap at opposite sides of the consecutive rings. Multiple SSRR approaches over SRR are preferred, as this creates a high electric field region between the resonator and microstrip line where a circular slot is created to be utilized for the sensing purpose. The electromagnetic simulations of the design and validation of the SSRR-based sensor structure are carried out in a CST Microwave Studio. Discrete ports are used for the excitation of the design and $S_{21}$ (transmission coefficient) is investigated, to evaluate the response of the sensor. The excitation through the microstrip line produces a high electric field region inside the substrate between the split-ring resonators and the microstrip line. This region becomes extremely sensitive to dielectric changes in its vicinity at resonance and will cause a shift in resonant frequency. The addition of more split-ring resonators results in an increase in the size of the overall structure and increases the capacitance, which, consequently, decreases the resonant frequency of the design. Additionally, the increasing number of split rings supplements the electric field localization between the resonators and the microstrip lines, hence enhancing the sensing capability of the design. The design parameters of the SSRR (the size of the outermost ring, the distance between the rings, the gap in each ring, and the width of each ring) also play a key role in influencing the capacitance and inductance of the resonators, which collectively govern their resonant frequency. The capacitive area in this sensing structure is the region where the maximum electric fields are concentrated. The placement of the sample is quite close to the territory of the maximum electric fields. The capacitance changes, due to the variations in the dielectric characteristics of the sample and the electric field distortion by the sample under test. The variation in capacitance is governed by changes in the dielectric properties of a sample and, consequently, a shift in the resonant frequency in the same fashion. Figure 6 shows PET based sensor design with equivalent circuit model. Therefore, the change in concentration ratio resulting in variation in the dielectric properties can be estimated through variations and shifts in the resonant frequency.

Similarly, the dielectric losses of the same material can be quantified by a change in the Q factor, given as [46]

(3) $$Q=\frac{\epsilon_{r}^{\prime }}{\epsilon_{r}^{\prime \prime }}$$

The sensitivity of such a metamaterial-based sensor can be calculated as given below [20]:

(4) $$S=\frac{f_{empty}-f_{sample}}{f_{empty}\left(\epsilon_{r}-1\right)}\times 100$$

Here, $f_{empty}$ and $f_{sample}$ are the resonant frequencies measured without sample and with sample, respectively, and S is the calculated sensitivity of the design.

## 3. Measurements

### 3.1. Measurement of Dielectric Properties

**Figure 7:**
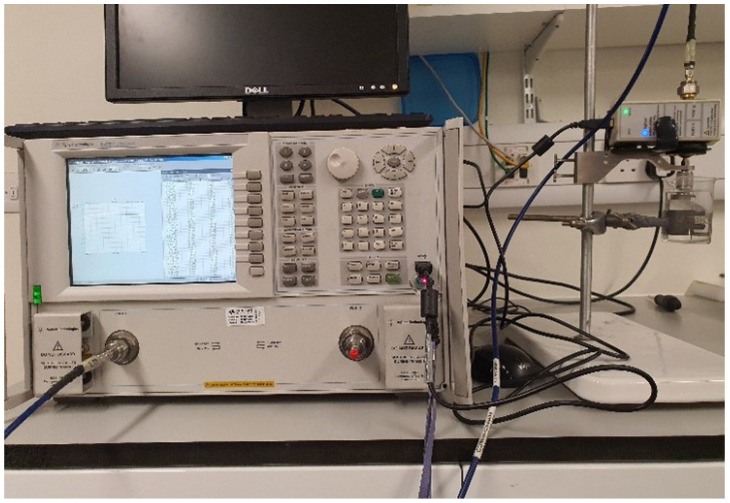
Measurement setup to compute the electrical permittivity of the prepared samples, using VNA and a dielectric probe kit.

The dielectric properties (electrical permittivity) of the samples were measured using a PNA-L (N5230C) vector network analyzer (VNA) with a frequency range of 10 MHz–20 GHz and Agilent 85070 dielectric probe kit. An Agilent 85062B kit was used for electronic calibration before measurements using references of short, open air, and distilled water at 25 °C. This calibration is very crucial and plays a vital role in accurate dielectric measurements [47]. Five samples of each ethanol and methanol solution were prepared, ranging from 10% to 50% concentration with a step size of 10%. Ethanol and methanol both had a purity of more than 99% when used in the preparation of the samples. Similarly, five samples of hydrocortisone (obtained from Sigma-Aldrich, St. Louis, MO, USA) were prepared, which were 10 mg, 20 mg, 30 mg, 40 mg, and 50 mg in 200 mL of 75% methanol aqueous solution. The dielectric properties were measured in the frequency range of 0.5–5 GHz, using a calibrated network analyzer and a dielectric probe kit. The complete measurement setup for the dielectric properties’ measurement is shown in Figure 7.

**Figure 8:**
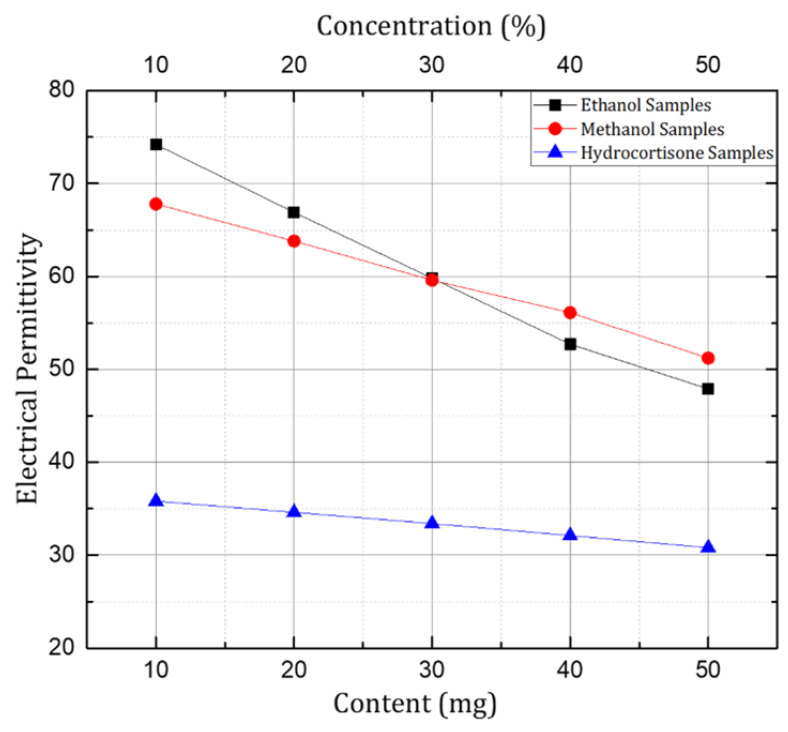
Variation in measured electrical permittivity of the prepared samples shown at 1.5 GHz.

The measured dielectric properties extracted from the VNA were converted to text files to be uploaded to the CST Microwave Studio Program used for the simulations. Using the data uploaded for each sample, new materials were defined in the simulation software. These newly defined materials were used as samples for the numerical simulation of the designed sensor shown in Figure 8.

### 3.2. Sensor Measurements

**Figure 9:**
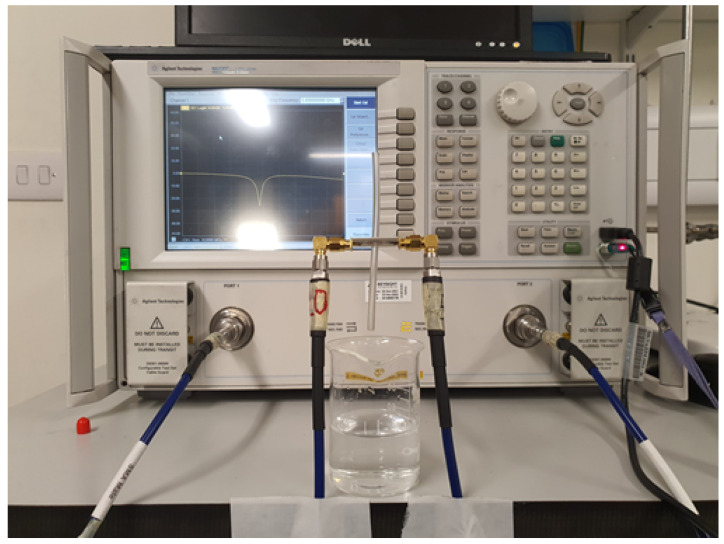
Fabricated design connected to vector network analyzer (VNA) for experimental measurements.

After a detailed investigation of the proposed sensor design in simulations, experimental measurements were carried out. Following the design fabrication, SMA (sub miniature version A) connectors were glued to prototypes with Rogers and PET substrate using conductive epoxy, allowing transverse electromagnetic (TEM) mode waves to propagate through the microstrip lines linked with the resonators. In TEM mode, the electric and magnetic field lines propagated transverse (perpendicular) to each other. All the samples were prepared again, as mentioned above, for experimental investigation. Pipettes and beakers were used for the accurate preparation of the liquid samples, while a highly accurate electronic weight scale (measurement accuracy of 1 μg) was used for the preparation of the hydrocortisone samples. All the equipment was washed and dried carefully after the preparation of every sample, to avoid any contamination. Initially, the VNA was calibrated in the frequency range of 0.5–5 GHz, using the Agilent 85062B kit. Two coaxial cables were connected to the sensor structure and the transmission coefficient ($S_{21}$) was measured using an electronically calibrated PNA-L network analyzer (N5230C). A glass capillary tube was placed at the center, inside the circular slot, where the samples were injected for experimental investigation. The liquid samples were injected from the top of the capillary tube and collected at the bottom, to avoid chemical spillage. Moreover, the absence of other electronic devices near the measurement setup was ensured, to minimize the possibility of any faulty measurements caused by electromagnetic interference. The temperature of the measuring environment and samples to be measured was maintained in the range of 20–25 °C, to ensure consistent measurements avoiding the effect of temperature. All the experimental measurements were repeatable and provided reliable outcomes for every measurement. The fabricated design and measurement setup (connected together for experimental investigation) is shown in Figure 9.

## 4. Resultsand Discussion

### 4.1. Chemical Sensing

The proposed flexible microwave sensor structure was designed, modeled, and numerically investigated in the CST Microwave Studio program, and experimental measurements were conducted separately for practical validation. The performance of the sensor structure was evaluated based on measuring the transmission coefficient ($S_{21}$). The sensor design structure was fabricated and investigated on three different substrates: Rogers RO4003C, Rogers RT/Duroid 5880, and flexible PET. In the case of ethanol and methanol, the selected range of concentration was 10–50% with a step size of 10%. In the case of the hydrocortisone solution, the selected range of hydrocortisone was 10–50 mg in 200 mL of 75% methanol solution.

**Figure 10:**
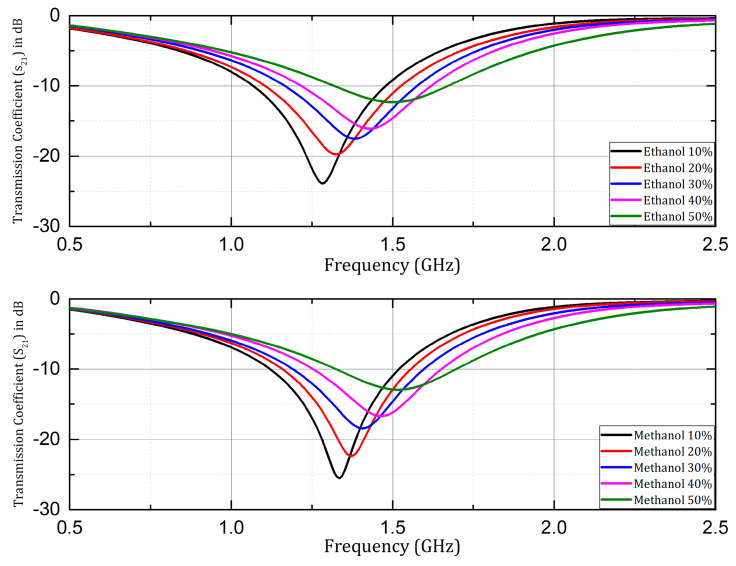
Measured transmission coefficient ($S_{21}$) when ethanol and methanol samples were investigated with prototype on Rogers RO4003C.

**Figure 11:**
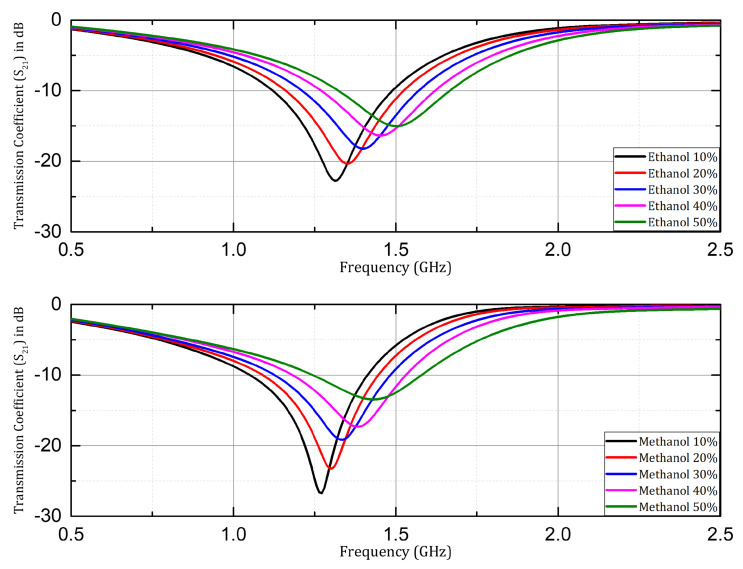
Measured transmission coefficient ($S_{21}$) when ethanol and methanol samples were investigated with prototype on Rogers RT/Duroid 5880.

The sensor structure with Rogers RO4003C substrate was investigated for microfluidic applications, using ethanol, and methanol samples in aqueous solutions. The transmission coefficient ($S_{21}$) was investigated, and is presented here with samples having concentrations of 10%, 20%, 30%, 40%, and 50% of ethanol and methanol. A significant shift in resonant frequency was observed when the concentration of the respective organic solvents (ethanol & methanol) was changed in the aqueous solution samples as shown in Figure 10. Similarly, in the case of the Rogers RT/Duroid 5880 substrate the resonant frequency increased with the increase in concentration by a step size of 10% in both cases of ethanol and methanol samples prepared in aqueous solutions as shown in Figure 11. Overall, in both cases of Rogers’ substrates, the trend of increasing resonant frequency with an increase in the concentration of organic chemicals (ethanol and methanol) remained the same throughout the experimentation process. The Q factor in the mentioned cases was non-linear with respect to change in concentration due to the non-linear loss function of the water mixture.

**Figure 12:**
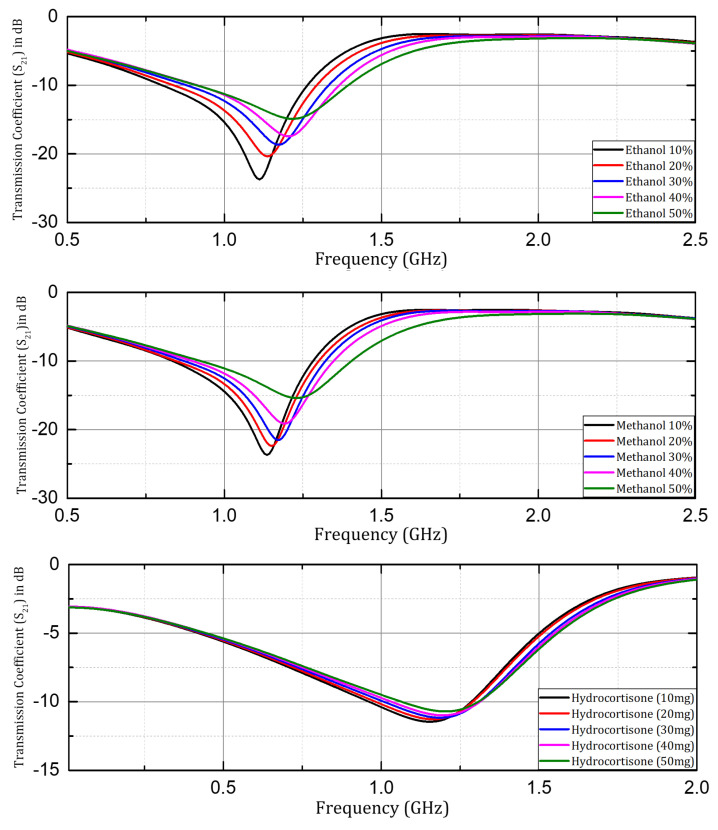
Measuredtransmission coefficient ($S_{21}$) when ethanol, methanol, and hydrocortisone samples were investigated with prototype on PET substrate.

After the investigation of prototypes with the Rogers non-flexible substrates, the sensor structure with the PET substrate was investigated for microfluidic applications using ethanol and methanol samples in aqueous solutions, and for healthcare applications hydrocortisone samples were investigated. The transmission coefficient ($S_{21}$) was investigated and is presented here with samples having concentrations of 10%, 20%, 30%, 40%, and 50% of ethanol and methanol, and samples of hydrocortisone (chemical derivative of human cortisol) with a quantity of 10mg, 20 mg, 30 mg, 40 mg, and 50 mg in 200 mL of 75% methanol solution. A significant shift in resonant frequency was observed when the concentration of respective organic solvents (ethanol and methanol) was changed in the aqueous solution samples. Similarly, in the case of the hydrocortisone samples the resonant frequency increased with an increase in the content of hydrocortisone, but it was not as significant as compared to the behavior shown by the organic solvent samples. The reason was that variation in the electrical permittivity is small with the change in the content of the hydrocortisone, as shown in Figure 12.

**Figure 13:**
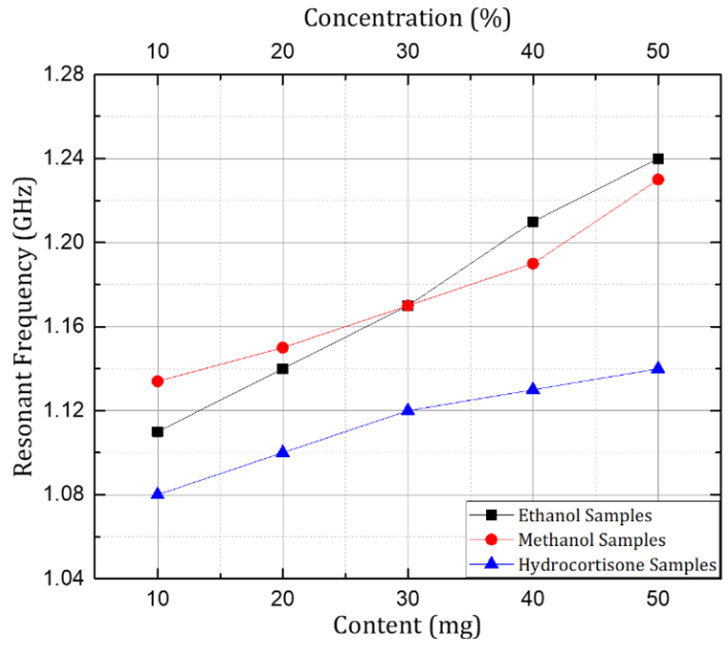
Graph showing variation in resonant frequency with changes in concentration of organic solvents (ethanol and methanol) and content of hydrocortisone in samples investigated with PET substrate.

In short, the resonant frequency increased with the increase in the concentration of ethanol, methanol, and hydrocortisone in their corresponding samples. The trend remained the same throughout the experimentation process when the concentration of ethanol and methanol was changed from 10% to 50%. Similar behavior would be observed for further concentrations (60–100%) as there was linear variation in the dielectric properties for the samples with higher concentrations. Similarly, the same behavior was depicted by the sensor when the concentration of hydrocortisone was varied from 10 mg to 50 mg in the respective solution samples. The linear variation of frequency versus the concentration of the mentioned chemicals is shown in Figure 13. In the case of the 30% ethanol and 30% methanol samples, the resonant frequency was the same, due to the fact that both samples had the same electrical permittivity at the given frequency range, also explained in Figure 8.

In the case of the flexible substrate, the linear variation in the resonant frequency was still perceptible with variations in the concentration of the ethanol and methanol, and also with variations in the content of the hydrocortisone in the provided samples. Therefore, the results of the PET-based prototype are promising when compared to and analyzed with the prototypes based on the Rogers substrates. So, it can be concluded that a flexible PET-based sensor design can replace and substitute non-flexible substrates without compromising on sensitivity while providing extra features of flexibility and conformability, making it ideal for biomedical applications and healthcare.

### 4.2. Flexible Applications

Flexible sensor structures are exposed to various stresses during bending at different angles along different planes, due to the nature of their usage for various chemical applications and healthcare. These sensors are conformably attached to different types of surfaces in the chemical industry and in the human body for medical applications [29]. Previous studies have noted that bending in the XZ-plane (E-plane) has a more important effect on the antenna resonance length than bending through the YZ-plane (H-plane) [30]. Therefore, in this study, the investigation of the sensor with bending effects was performed by bending the sensor structure along the XZ-plane around a cylinder with diameters of 40 mm, 50 mm, 70 mm, and 150 mm (70 mm representing the upper human limb and 150 mm representing the lower human limb), where bending radius refers to the radius of the cylinder on which the sensor design was attached and bent to various degrees. Between the two sensor designs based on PET and the Rogers 5880 substrate, PET is a better option than Rogers 5880 for flexible applications, due to being cost-effective, having better dielectric strength, high electrical stability, and better tensile strength and elasticity [48].

**Figure 14:**
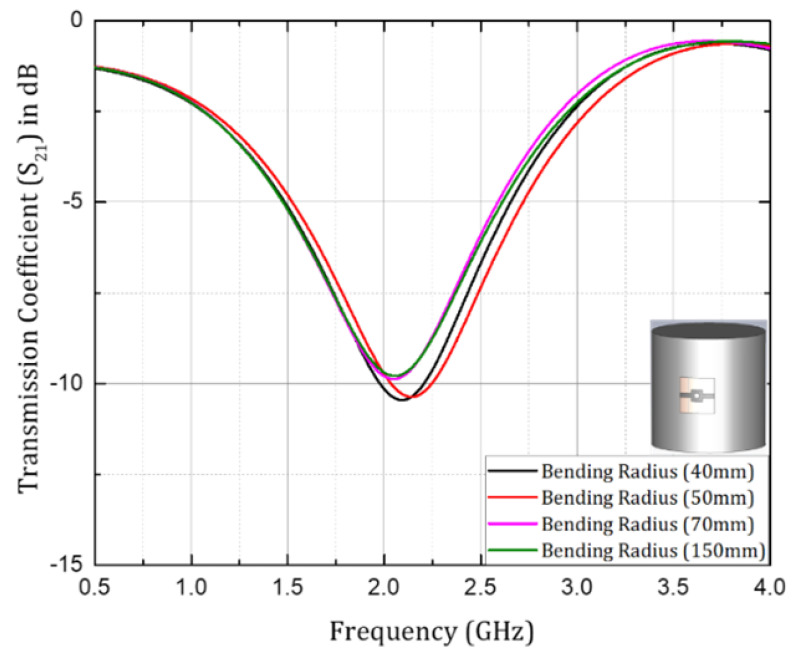
Variation in resonant frequency with increase in the bending radius of the cylinder on which the proposed design was bent over PET flexible substrate.

The sensor structure under investigation could be attached to the cylinder using Kapton tape for experimental measurements, to avoid extra physical stress on the connectors delicately bound using conductive epoxy. Variations in resonant frequency with change in the bending radius (the radius of the cylinder on which the sensor has been bent for investigation) in the transmission coefficient ($S_{21}$) are depicted in Figure 14. The graph shows that the resonant frequency was quite similar in the case of the 70 mm and 150 mm bending radius. This was due to the fact that when the bending radius was increased beyond a certain level the structure started behaving like a planar structure and the response became consistent.

A very small difference in resonant frequency was observed when the bending effect was introduced into the design, and it remained consistent when the bending radius was varied in the range of 40 mm to 150 mm, making it practical and suitable for flexible applications. Therefore, flexible sensor design can substitute sensors based on non-flexible substrates without compromising on sensibility. Moreover, such designs are highly desirable for healthcare applications, due to features like conformability and flexibility [49,50]. Progression in this field will facilitate implementations of more reliable sensors in the future, hence providing an extensive solution for non-invasive and contactless measurements in chemical and biomedical applications. Table 1 compares the proposed sensor with state-of-the-art microwave sensing devices used for microfluidic applications.

## 5. Conclusions

Electromagnetic resonators and structures provide an appealing solution for microfluidic applications with sensors having high sensitivity for concentration estimation and detection of various chemicals and biochemicals. In this article, an economical, compact, contactless, and flexible electromagnetic sensor was presented, which was used to detect and estimate variation in concentrations of chemicals (ethanol and methanol) and biochemicals (hydrocortisone – a chemical derivative of cortisol, a biomarker of stress and cardiovascular effects). The sensor design showed the consistent behavior of a significant increase in resonant frequency in response to an increase in the concentration of ethanol, methanol, and hydrocortisone in the prepared samples. In the case of the ethanol and methanol, a shift in resonant frequency of more than 50 MHz was observed with a change in concentration by 10%, whereas in the case of hydrocortisone the shift was smaller, due to less variation in electrical permittivity with an increase in concentration. The proposed flexible PET-based sensor is promising, as it showed a maximum sensitivity value of 0.654, which was quite decent in comparison to its non-flexible counterparts. Therefore, a flexible PET-based sensor design can replace and substitute non-flexible substrates without compromising on sensitivity while providing extra features of flexibility and conformability, making it ideal for biomedical applications and healthcare. It can be concluded that the proposed sensor design is promising for microfluidic measurements while offering a convenient and low-cost operation.

## Figures and Tables

**Table 1:** Comparison of the proposed sensor with state-of-the-art microwave sensing devices used for microfluidic applications.

Freq. (GHz)	Substrate	Liquid Flow Mechanism	Maximum Sensitivity	Fabrication	Flexibility	Cost	Reference
2.4	FR-4	Capillary tube	0.214	Easy	No	Low	[20]
2	RT6002	PDMS	0.436	Complex	No	High	[36]
1	RO3010	PET film	0.195	Complex	No	High	[35]
5.8	F4B-2	Submersible	0.102	Moderate	No	Moderate	[38]
2.45	RO4003C	Capillary tube	0.643	Easy	No	Moderate	This work
2.8	RT5880	Capillary tube	0.734	Easy	No	Moderate	This work
2.2	PET	Capillary tube	0.654	Easy	Yes	Low	This work

## Data Availability

The original data presented in the study are openly available.
